# Supplementary material for: Mechanisms governing the pioneering and redistribution capabilities of the non-classical pioneer PU.1
Source: Nat Commun. 2020 Jan 21;11:402. doi: 10.1038/s41467-019-13960-2 (PMC6972792; doi:10.1038/s41467-019-13960-2)
Supplement: Supplementary file 7 — Source data [file 41467_2019_13960_MOESM7_ESM.zip › Source_Data/FigureS5/FigureS5A_MotifsCommonPeaks/homerResults/motif39.similar.html]

motif39

## Information for motif39

G
T
C
A
T
A
G
C
A
G
C
T
T
C
G
A
A
G
T
C
T
C
G
A
C
G
T
A
G
A
C
T
A
C
G
T
G
A
T
C
A
G
T
C
G
A
T
C
  
Reverse Opposite:  

C
T
A
G
C
T
A
G
C
T
A
G
T
G
C
A
C
T
G
A
G
C
A
T
A
G
C
T
C
T
A
G
A
G
C
T
T
C
G
A
A
T
C
G
C
A
G
T
  

|  |  |
| --- | --- |
| p-value: | 1e-30 |
| log p-value: | -7.135e+01 |
| Information Content per bp: | 1.723 |
| Number of Target Sequences with motif | 158.0 |
| Percentage of Target Sequences with motif | 1.78% |
| Number of Background Sequences with motif | 178.3 |
| Percentage of Background Sequences with motif | 0.47% |
| Average Position of motif in Targets | 105.2 +/- 56.7bp |
| Average Position of motif in Background | 94.5 +/- 71.1bp |
| Strand Bias (log2 ratio + to - strand density) | 0.0 |
| Multiplicity (# of sites on avg that occur together) | 1.17 |
| Motif File: | file (matrix) reverse opposite |

### Similar de novo motifs found

|  |  |  |  |  |  |  |  |
| --- | --- | --- | --- | --- | --- | --- | --- |
| Rank | Match Score | Redundant Motif | P-value | log P-value | % of Targets | % of Background | Motif file |
| 1 | 0.954 | C G T A T G A C G A C T G T C A G A T C T G C A C G T A G A C T A C G T A T G C G A T C A G T C C T G A A C G T | 1e-27 | -64.165536 | 1.70% | 0.47% | motif file (matrix) |
| 2 | 0.931 | G T C A G T A C G A C T T G C A A G T C T C G A G T C A G A C T C A G T A G T C G A T C | 1e-26 | -60.000020 | 2.04% | 0.69% | motif file (matrix) |
| 3 | 0.982 | T C G A G A T C A G C T G T C A G A T C T C G A C T G A G A C T A G C T A G T C G T A C G T A C T C A G | 1e-24 | -56.121080 | 1.69% | 0.52% | motif file (matrix) |
